# Unraveling the hydration dynamics of ACC_1–13_K_24_ with ATP: From liquid to droplet to amyloid fibril

**DOI:** 10.1016/j.bpj.2024.09.011

**Published:** 2024-09-11

**Authors:** Sampad Bag, Robert Dec, Simone Pezzotti, Rudhi Ranjan Sahoo, Gerhard Schwaab, Roland Winter, Martina Havenith

**Affiliations:** 1 Physical Chemistry-II, Ruhr-University Bochum, Bochum, Germany; 2 Physical Chemistry I - Biophysical Chemistry, Department of Chemistry and Chemical Biology, TU Dortmund University, Dortmund, Germany; 3 National Institute of Science Education and Research, Bhubaneswar, India

## Abstract

In order to achieve a comprehensive understanding of protein aggregation processes, an exploration of solvation dynamics, a key yet intricate component of biological phenomena, is mandatory. In the present study, we used Fourier transform infrared spectroscopy and terahertz spectroscopy complemented by atomic force microscopy and kinetic experiments utilizing thioflavin T fluorescence to elucidate the changes in solvation dynamics during liquid-liquid phase separation and subsequent amyloid fibril formation, the latter representing a transition from liquid to solid phase separation. These processes are pivotal in the pathology of neurodegenerative disorders such as Alzheimer’s and Parkinson’s diseases. We focus on the ACC_1–13_K_24_-ATP protein complex, which undergoes fibril formation followed by droplet generation. Our investigation reveals the importance of hydration as a driving force in these processes, offering new insights into the molecular mechanisms at play.

## Significance

Protein aggregation is a well-known phenomenon that plays a crucial role in various biological processes, such as transcription, ribosome biogenesis, and cellular signaling, and is also implicated in numerous protein-related diseases (20). The aggregation process is highly dependent on the microenvironment of the proteins. The research presented in this paper addresses a critical gap in our understanding of the solvation thermodynamics involved in the liquid-liquid phase separation and liquid-solid phase separation processes. Utilizing advanced terahertz-Fourier transform infrared spectroscopy, we have provided novel insights into the changes in hydration water structure during these phase transitions. This study provides significant insights into how the hydration water structure evolves as the protein structure transitions from a homogeneous solution to droplets and eventually to fibrils over time. Additionally, our findings elucidate how the aggregation process is facilitated both entropically and enthalpically by different hydration water structures.

## Introduction

In the complex ballet of cellular dynamics, the phenomena of amyloid fibril formation and liquid-liquid phase separation (LLPS) have emerged as vital actors in both physiological processes (1,2) and pathological conditions (3,4). Interestingly, the LLPS phenomenon can be linked to the initial stages of amyloidogenesis. Among the disorders frequently linked to amyloids, Alzheimer’s disease, Parkinson’s disease, and systemic amyloidosis are noteworthy examples. Conversely, so-called functional amyloids, such as Pmel17 involved in melanin synthesis in the skin (5), curli fibers responsible for biofilm formation in bacteria (6), or yeast prions like Sup35 that participate in controlling genetic expression (7), highlight the beneficial aspects of some other amyloids. Thus, the exceptional mechanical strength and stability of amyloid fibers can be destructive to cells but are also ingeniously utilized by living organisms.

LLPS, a process by which a homogenous molecular solution separates into two distinct liquid phases, is recognized for its crucial role in the spatial organization of biological matter and facilitates the regulation of biochemical reactions (8,9) and cellular architecture (10) without the need for membrane-bound organelles. Emerging through LLPS, classic examples of membrane-less organelles (also known as biomolecular condensates) are stress granules, which help in mRNA regulation under stress conditions (11), and P-bodies, involved in mRNA degradation and storage (12). Nowadays, many examples of proteins and peptides are known where amyloidogenesis is preceded by the formation of liquid protein condensates (13,14,15,16). However, the mechanisms underlying LLPS and amyloid formation are still far from being fully understood. In particular, the role of water molecules in these processes, which constitute the milieu for the morphological and structural conversions occurring, remain inadequately explored.

In this study, we focused on the unexplored water contribution to amyloid formation preceded by LLPS by combining terahertz (THz)-calorimetry, turbidity measurements, fluorescence spectroscopy, optical microscopy, and atomic force microscopy (AFM). While fluorescence and microscopy allow us to determine the formation of liquid condensates and characterize the subsequently formed amyloid fibrils, THz-calorimetry directly connects spectroscopic to solvation thermodynamics quantities, allowing us to characterize the accompanying changes in hydration water and the impact that solvation free energies have on the thermodynamics of the process.

We focus our investigation on the ACC_1–13_K_24_-ATP system. ACC_1–13_K_24_ is a synthetic peptide comprising the N-terminal fragment of the insulin A chain (13 residues long), identified in earlier research as a highly amyloidogenic fragment of this protein (17,18). The isolated ACC_1–13_ fragment readily forms amyloid, which happens without the involvement of LLPS (18). The K_24_ fragment is a polylysine chain (24 residues long) covalently attached to the C-terminus of the ACC_1–13_ fragment. The idea to extend the ACC_1–13_ peptide with a polylysine chain was inspired, inter alia, by an article by Koga et al. (19), wherein the authors demonstrate that, mixed together, polylysine and ATP undergo LLPS to form droplets. Within these polylysine-ATP droplet complexes, positively charged lysine residues are electrostatically balanced by the negative charged ATP molecules. At a pH close to physiological value, due to the highly charged K_24_ fragment, the ACC_1–13_K_24_ peptide is no longer amyloidogenic, nor does it undergo LLPS. However, the addition of an appropriate amount of ATP, similar to the case of polylysine, balances this charge, leading to the formation of droplets, and then (and this time different from the polylysine case), thanks to the presence of the ACC_1–13_ fragment, amyloid is also formed (20,21).

## Materials and methods

### Sample preparation

The ACC_1–13_K_24_ peptide was custom synthesized by Biosynth (Staad, Switzerland) and provided as a trifluoroacetic acid salt. All additional reagents, including ATP and poly-L-lysine hydrobromide, were sourced from MilliporeSigma (Burlington, MA, USA). The peptide was dissolved in deionized water, and the solution’s pH was adjusted to 6.5 using diluted sodium hydroxide (NaOH). A freshly made ATP solution was used to prepare the samples, with its pH adjusted in the same manner. The ready-to-measure sample consisted of ACC_1–13_K_24_ at a concentration of 1 mg/mL and ATP at a ratio ensuring 1 ATP molecule per 3 lysine residues. For the kinetic experiments utilizing thioflavin T (ThT) fluorescence, the samples contained the fluorophore at a concentration of 20 *μ*M. Samples containing 1 mg/mL of poly-L-lysine instead of ACC_1–13_K_24_ peptide were prepared in an analogous manner and maintained the same ATP/lysine ratio.

### FT-IR/THz spectroscopy

The spectra were acquired using a Fourier transform infrared/THz (FT-IR/THz) spectrometer (Vertex 80v, Bruker, Billerica, MA, USA), equipped with a mercury vapor lamp source and a helium-cooled silicon bolometer detector (Infrared Laboratories, Tucson, AZ, USA), covering the spectral range of 30–650 cm^−1^. The FT-IR/THz sample compartment featured a single-reflection attenuated total reflectance (ATR) unit (MVP-Pro, Harrick Scientific, Pleasantville, NY, USA) housing a temperature-controlled diamond crystal (500 mm in diameter, Harrick Scientific). ATR absorption spectra (*α* (*ν*)) were determined as follows:

(1) $$\alpha \left(\nu \right)=-\frac{1}{d_{p}}\ln\left(\frac{I\left(\nu \right)}{I_{0}\left(\nu \right)}\right)$$

(2) $$d_{p}=\frac{\lambda }{2\pi \sqrt{{\;n}_{\text{diamond}}^{2}{\;\sin\left(\Theta \right)}^{2}-n_{\text{sample}}^{2}}}$$

*I*(*ν*) and *I*_0_(*ν*) denote the frequency-dependent intensities of the sample and reference, respectively. The reference spectrum was recorded after cleaning the diamond surface. In scenarios involving strongly absorbing samples, like aqueous solutions, the electric field decay at the interface becomes complex, introducing a theoretical upper limit for the penetration depth, denoted as *d*_*p*_, which defines the extent to which the evanescent wave can propagate into the sample. The refractive index of the diamond (*n*_diamond_) is 2.38, and for an aqueous sample, we approximated a frequency-independent refractive index (*n*_sample_) of 1.5. Liquid-liquid phase separated intrinsically disordered protein condensates typically exhibit protein concentrations in the range of hundreds of milligrams per milliliter. These protein condensates retain a significant amount of water (8,9). Therefore, we assumed that the refractive index of the sample is the same as those for aqueous samples. At the beginning of the measurement, we conjoined protein solutions atop the ATR crystal, initiating droplet formation at a given temperature. The resulting condensed protein-rich phase settled onto the ATR crystal. With increasing time, we observed more and more protein condensates at the diamond solution interface. These were probed in the THz-FT-IR frequency range by the evanescent field, and spectra were recorded as a function of time.

### LLPS and fibrillization kinetics

The kinetics of LLPS and amyloidogenic fibrillization was monitored using black 96-well microplates with transparent bottoms, utilizing an Infinite M200 plate reader from Tecan (Männedorf, Switzerland). Changes in optical density at 500 nm were recorded to track the progression of LLPS. Amyloidogenic fibrillization kinetics was assessed through the measurement of ThT fluorescence intensity, with an excitation wavelength of 440 nm and emission detected at 485 nm. Each well was filled with a 50 *μ*L sample of freshly prepared peptide-ATP solution containing ThT. Unless specified otherwise, measurements were conducted at 37°C with gentle agitation set at 300 rpm.

### AFM

Aqueous suspension of the ACC_1–13_K_24_-ATP coaggregate, previously washed to remove excess codissolved salts, was further diluted with deionized water by a factor of eight. A 10 *μ*L aliquot of diluted suspension was deposited onto a freshly cleaved mica surface and allowed to air dry overnight. AFM tapping-mode measurements were conducted employing a NanoScope IIIa system from Veeco Instruments (Plainview, NY, USA), equipped with Tap300Al-G sensors (300 kHz, 40 N/m) sourced from BudgetSensors (Sofia, Bulgaria). Image analysis was performed using the NanoScope Analysis software.

### ATR FT-IR spectroscopy

Aqueous suspensions of the ACC_1–13_K_24_-ATP coaggregate were washed with deionized water and deposited on the diamond surface of a single-reflection ATR accessory for the Nicolet iS50 FT-IR spectrometer from Thermo Fisher Scientific (Waltham, MA, USA). To acquire a single ATR FT-IR spectrum, we recorded 32 interferograms at a nominal resolution of 2 cm^−1^. The spectra were analyzed using the GRAMS software from Thermo Fisher Scientific.

### Optical microscopy

Imaging of the ACC_1–13_K_24_-ATP droplets and mature aggregate was performed using an Eclipse TE2000-U microscope (Nikon, Melville, NY, USA) equipped with a Nikon Plan Fluor 20× objective lens (NA 0.45, WD 7.4). This setup was further enhanced by an X-Lite-120 fluorescence illumination system (EXFO) and an F41-025 HQ-Set P-GFP BP filter, specifically for ThT fluorescence imaging. Samples were deposited onto microscope slides for analysis. For monitoring the transformation from ACC_1–13_K_24_-ATP droplets to amyloid aggregates, the same setup was utilized, with the addition of a thermostat to maintain a constant temperature of 37°C. Image analysis was performed using ImageJ software.

## Results and discussion

**Figure 1:**
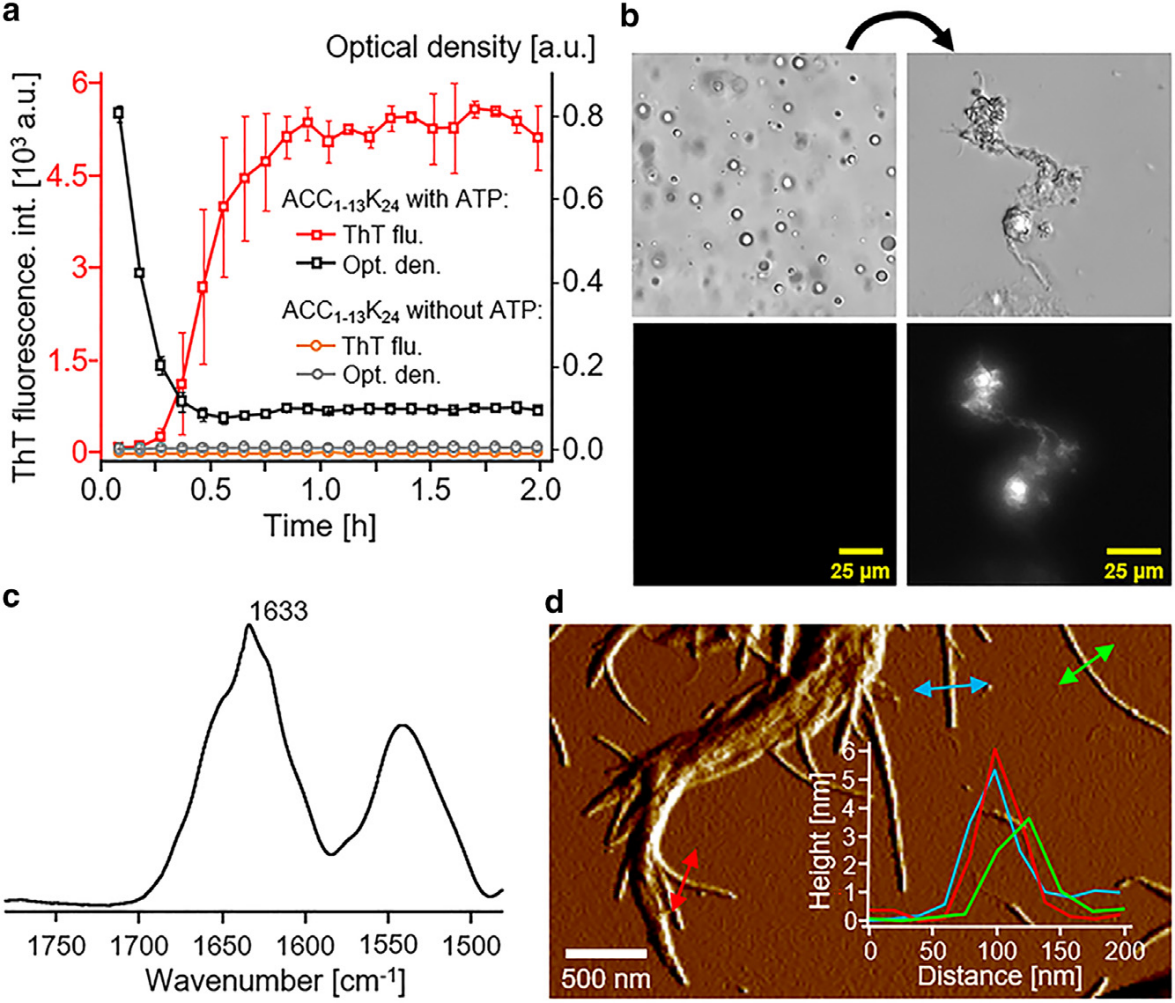
ACC_1–13_K_24_ peptide forming an amyloid coaggregate with ATP, the formation of which is preceded by LLPS. (*a*) Kinetics of changes in ThT fluorescence intensity (*red and orange trajectories*) and optical density (*black and gray trajectories*) measured for a freshly prepared sample containing ACC_1–13_K_24_ peptide and ATP and a control sample containing the peptide but no ATP (1 mg/mL ACC_1–13_K_24_, 1 ATP per 3 lysine residues [if used], 20 *μ*M ThT, H_2_O [pH 6.5]; gentle shaking, 37°C). (*b*) Sample containing ACC_1–13_K_24_ peptide and ATP prepared as in case (*a*) observed by light microscopy (observations made at room temperature, no shaking) under normal light (*top*) or ThT fluorescence excitation light (*bottom*) shortly after mixing the peptide with ATP (*left*) or for the mature ACC_1–13_K_24_-ATP coaggregate (*right*). (*c*) FT-IR-ATR spectrum in the range of the amide I and amide II bands measured for the washed, mature ACC_1–13_K_24_-ATP coaggregate. (*d*) Amplitude AFM image of the washed, mature ACC_1–13_K_24_-ATP coaggregate. The inset shows height profiles for several selected fibrils, obtained from the corresponding height data.

In Fig. 1, we summarize the results of the structural characterization of the ACC_1–13_K_24_-ATP mixture upon LLPS and fibrillization. Fig. 1 *a* shows the results of kinetic measurements of ThT fluorescence intensity and optical density, recorded for a sample containing ACC_1–13_K_24_ peptide and ATP (and also for a control sample containing the peptide but no ATP). For the fresh mixture of ACC_1–13_K_24_ peptide and ATP, a very rapid, sigmoidal increase in ThT fluorescence intensity was observed, indicating that the amyloidogenesis process was ongoing. The amyloid nature of the mature ACC_1–13_K_24_-ATP sample was validated by results from FT-IR-ATR spectroscopic (Fig. 1 *c*) and AFM (Fig. 1 *d*) analyses. The shape and peak wavenumber (1633 cm^−1^) of the amide I′ vibrational band revealed that a parallel intermolecular β sheet structure predominates as the main secondary structural element in the centrifuged and washed ACC_1–13_K_24_-ATP coaggregate. No band was observed beyond 1680 cm^−1^, which confirms the lack of an antiparallel β sheet structure. Subsequently, in the AFM image obtained for the same mature ACC_1–13_K_24_-ATP coaggregate, fibrillar structures with dimensions typical of amyloid fibers were visible. Yet, very importantly, and as shown by the bright-field microscopy image in Fig. 1 *b* (*left*), which was recorded within minutes of sample preparation of the ACC_1–13_K_24_ peptide and ATP, fibrillation was preceded by LLPS. The condensed droplets forming in the ACC_1–13_K_24_ + ATP system were not ThT active, yet they scatter light very strongly, which is visible on the trajectory of optical density (i.e., turbidity) changes in Fig. 1 *a*. Within 30 min, these droplets transformed into less light-scattering but now ThT-active amyloid aggregates (Fig. 1 *b*, *right*).

**Figure 2:**
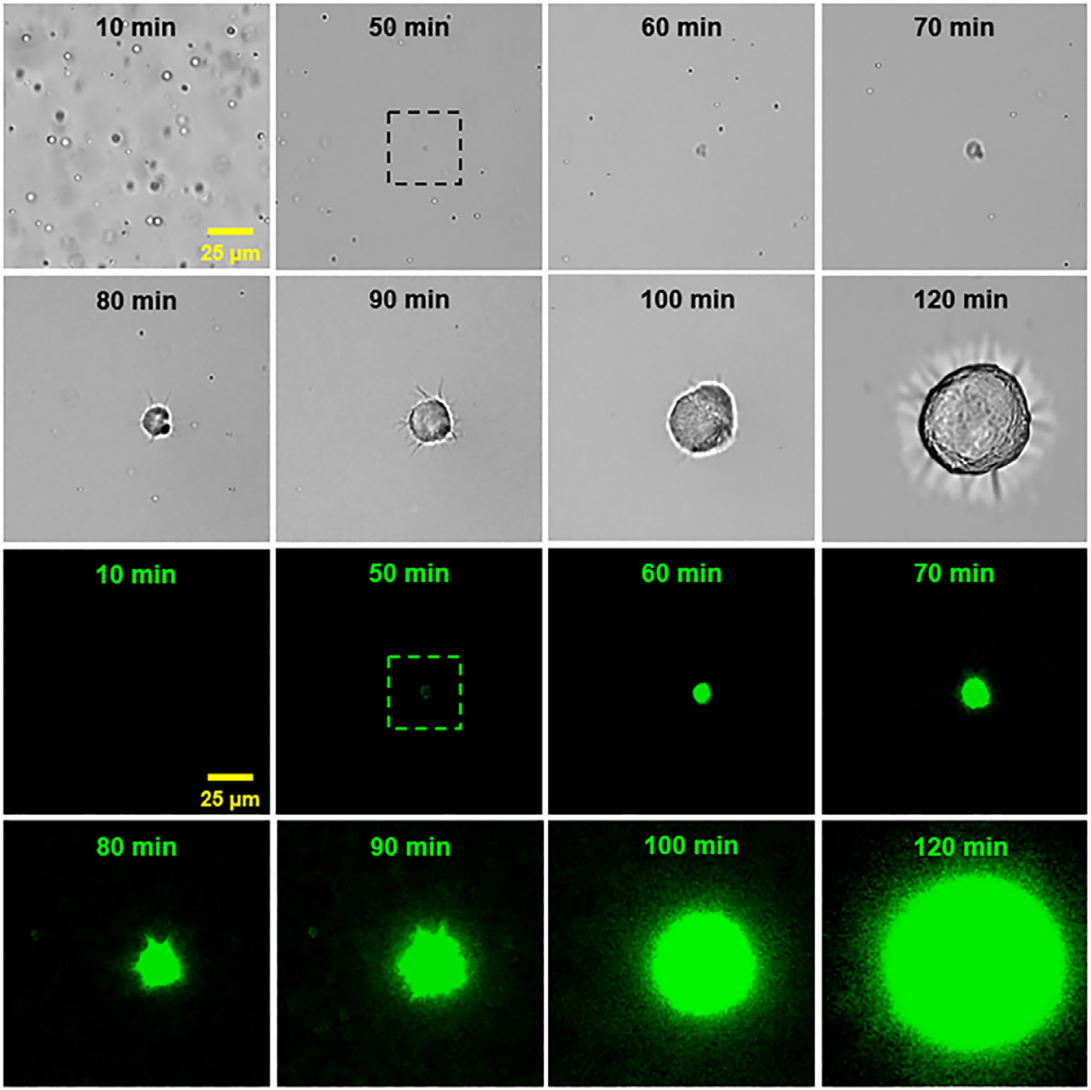
Transition from ThT-inactive ACC_1–13_K_24_-ATP droplets into ThT-active amyloid aggregates, visualized by bright-field optical microscopy. Observations under visible light (*top*) and ThT fluorescence excitation light (*bottom*) for an incubated sample containing ACC_1–13_K_24_ peptide and ATP (1 mg/mL ACC_1–13_K_24_, 1 ATP per 3 lysine residues, 20 *μ*M ThT, H2O [pH 6.5]; no shaking, 37°C). The box with the dotted line (images marked “50 min”) indicates the first appearance of an aggregate, as observed in fluorescence as a bright spot.

The transition of ThT-inactive ACC_1–13_K_24_ droplets into ThT-active aggregates, shown in Fig. 1 *a*, is better illustrated in Fig. 2. The images presented in Fig. 2 were captured at differently matured samples containing ACC_1–13_K_24_ peptide and ATP. As visualized by optical bright-field microscopy, we observed a transition from ThT-inactive ACC_1–13_K_24_-ATP droplets into ThT-active amyloid aggregates. In the first phase, the number of droplets and droplet-shaped aggregates decreased, while the ThT assay still showed a relatively weak, but already recordable, affinity (images marked with “50 min”). These aggregates then rapidly increased in size, indicated by a stronger fluorescence of ThT. On the periphery of the aggregates, microscopic folds of fibrils were starting to be visible (images marked with “80 min” and “90 min”). As shown in the work by Dec et al. (20), where the conversion of ACC_1–13_K_24_ complexes from droplets to amyloid was studied by AFM, the amyloid fibrillization process sets in on the surface of still-existing droplets, which indicates that the conversion of the droplet to amyloid is indeed direct.

Based on the optical density measurements, we conclude that droplet formation starts instantaneously when the ACC_1–13_K_24_ peptide and ATP solutions are mixed, while the onset of amyloid formation takes place at a later time. As the droplets evolve into fibrils, these form parallel β sheet structures. The fibril formation was characterized by AFM (Fig. 1 *c*).

In the THz measurements, we focused on two distinct THz absorption bands, corresponding to water populations hydrating hydrophilic and hydrophobic groups. They provide information to delineate the thermodynamics of solvation for both hydrophobic and hydrophilic interactions during LLPS. Previously, we introduced the concept of THz-calorimetry, which explored the partial contributions of the hydration water contributions to the LLPS process in a two-step scenario (22,23). Initially, there is a Gibbs free energy expenditure (Δ*G*_cavity_) associated with the formation of a solute cavity within the aqueous environment. Surrounding this cavity, water molecules form a quasi-two-dimensional network of hydrogen bonds, constituting the “cavity-wrap” population. The amplitude of the cavity-wrap band in the differential spectra (Δ*α*_wrap_) was found to be directly proportional to Δ*G*_cavity_. A change in the amplitude of this water population (as formed near hydrophobic patches) indicates a change in either the number of hydration water molecules of this cavity-wrap population or their spatial arrangement, both contributing to (Δ*G*_cavity_).

The presence of hydrophilic interactions implies that the interaction between solute and water exceeds those between water and its neighbor, resulting in a gain in free energy (Δ*G*_bound_), which serves to offset the energetic expense associated with creating a cavity in the solvent. For solutes that are predominantly hydrophobic and hence engage only weakly with water, Δ*G*_bound_ is largely driven by enthalpic contributions. This population is most sensitively probed in the frequency range of the librational mode, i.e., between 400 and 600 cm^−1^. Any sterical hinderance due to strong hydrogen bonds will result in a loss of water molecules in the frequency part probing the less hindered water, e.g., the softer librations around 400 cm^−1^, and an increase in the number of water molecules that are experiencing a stronger hinderance (compared to bulk water), e.g., the stiffer libations above 500 cm^−1^ (24,25).

**Figure 3:**
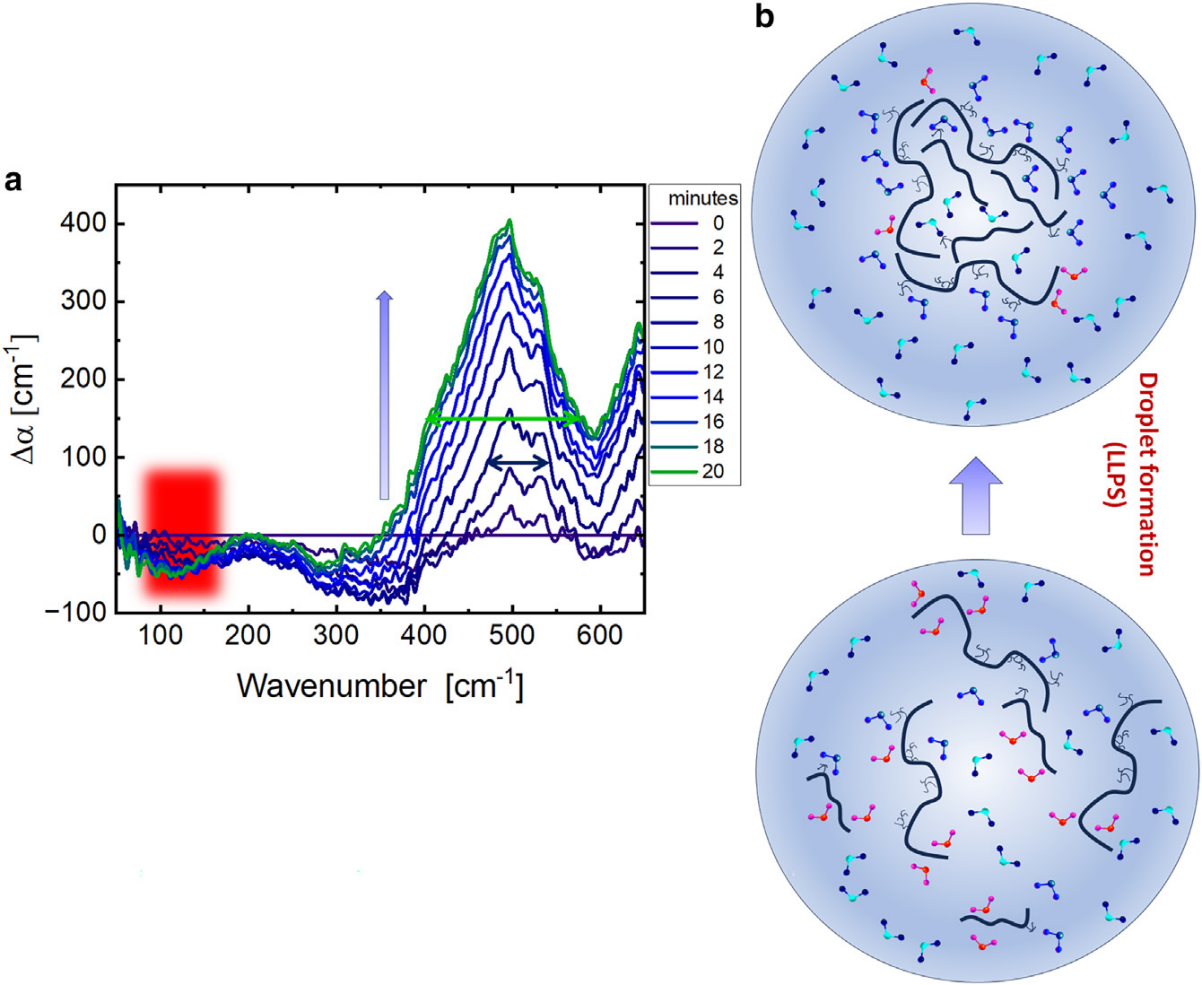
Terahertz fingerprint and schematic illustration of the LLPS process of the ACC_1–13_K_24_ and ATP mixture: (*a*) time series of Δ*α* spectra of the ACC_1–13_K_24_ (1 mg/mL) combined with an ATP solution (0.71 mg/mL) by subtracting the initial absorption spectra (*t* = 0). The time series is shown here for the first 20 min with an interval of 2 min where we see the droplet formation. The error is on the order of 1%. Around 100–150 cm^−1^, we record a decrease in intensity highlighted with a red-shaded area. This is attributed to a loss of more weakly bound cavity-wrap water hydrating the hydrophobic parts of the protein. The peak centered at 500 cm^−1^ shows an increase in intensity and a broadening in linewidth. The narrow features are attributed to a coupled ATP protein peak. Via coupling of the ATP, protein and hydration water, we observe a considerable line broadening (*b*). The schematic (not generated from computational simulation) illustrates the formation of droplets from the ATP, protein-solvent mixtures (LLPS). In this depiction, red water molecules signify cavity-wrap hydration water, dark blue denotes bound hydration water, and the bicolor light blue-dark blue molecules indicate bulk water. Upon LLPS, we observe a decrease of the wrap water population and an increase in bound water while the liquid condensate (droplet) is formed.

Here, THz absorption spectra were collected every 2 min over a 1 h period with a spectral resolution of 2 cm^−1^. For further analysis, we plot the spectral changes subsequent to initiation of ACC_1–13_K_24_ and ATP aggregation by subtracting the initial absorption spectra (*t* = 0) from subsequent spectra at time *t*. In Fig. 3 *a*, the Δ*α*(*t*) spectrum series elucidates the droplet formation (LLPS). We observed two prominent spectral changes within the first 20 min: a decrease in amplitude around 150 cm^−1^ and a broadening of the absorption mode centered at 500 cm^−1^. As was shown in our previous studies (22,24,26), a decrease in absorption, or negative Δ*α* centered at 150–160 cm^−1^, is indicative of a loss of an entropically unfavorable water population hydrating hydrophobic patches of the protein prior to protein condensation. Upon LLPS, this water population is released into the bulk liquid, which is entropically favorable.

We observed a second major spectral change in the frequency range between 400 and 550 cm^−1^, see Fig. 3. In the first 20 min, the absorption Δ*α* was increasing; in addition, a line broadening was observed. Both were found to be correlated with droplet formation. The underlying sharp peak centered at 500 cm^−1^, which is visible already at early times, cannot unambiguously be assigned. Compared to water modes, intramolecular modes are smaller in linewidth, corresponding to large amplitude motions. Any protein mode will increase upon droplet formation due to the enrichment of protein density in the condensate compared to the dilute protein solution (24,27). It can also be a coupled ATP-protein mode, which could also be enhanced when ATP binds to the protein. The line broadening is attributed to the relative increase of a coupling to the water network modes in the same frequency range, which is expected to increase the linewidth. While we are unable to dissect the observed spectrum in its distinct parts in the present paper, this was done in a joint experimental/simulation study on the solvated amino acid glycine, revealing a narrower intramolecular mode and a broader collective mode of the glycine water complex (28). The broad linewidth indicates that the polar groups of the proteins remain hydrated within the condensate, yet couple to the hydration water network modes. This is an essential prerequisite to keep the droplets in a liquid, reversible state (29). Observation of a coupled protein hydration water mode is expected if the water molecules are hydrogen bounded to hydrophilic groups (“bound water”). In Fig. 3 *b*, we give a schematic illustration (not generated by computational simulation), based on our interpretation, depicting the molecular mechanism of droplet formation. Whereas the entropically unfavorable hydration water forming a cavity around the hydrophobic groups is released into the bulk, the hydrophilic groups retain the hydration water (23). In the scheme, cavity-wrap and bound water molecules are represented by red and blue colors, respectively.

**Figure 4:**
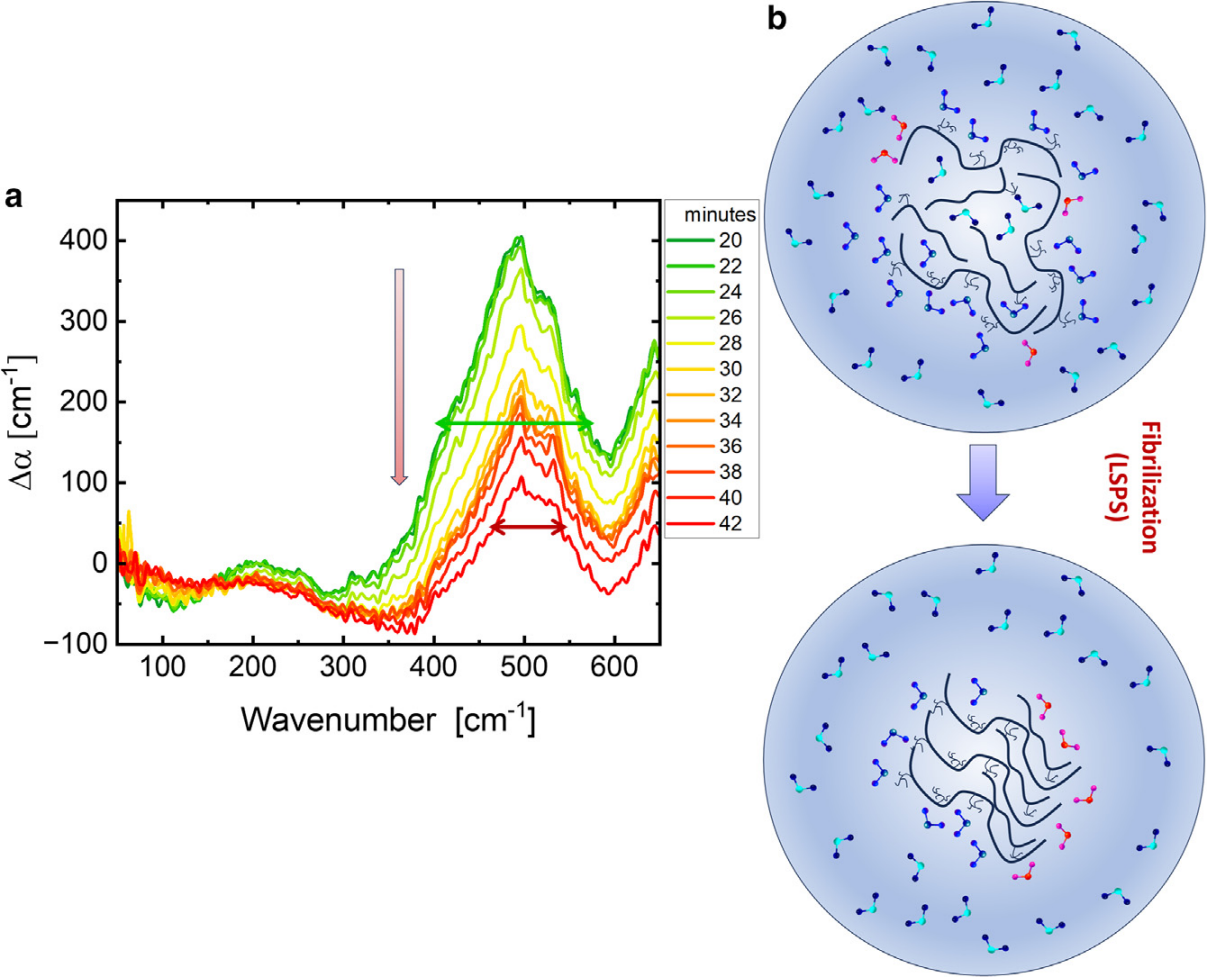
Series of THz spectra upon liquid-solid phase separation of the ACC_1–13_K_24_-ATP mixture: (*a*) Time series of difference (Δ*α*) spectra of ACC_1–13_K_24_ peptide (1 mg/mL) solvated in an ATP solution (0.71 mg/mL). Each spectrum shows the change in absorption at a given time point (*t*) compared to the initial absorption spectrum of the diluted mixture (*t* = 0). The error is on the order of 1%. We display here the spectra in the time interval between 20 and 42 min, i.e., when we observe liquid-liquid phase separation (LLPS) and subsequently the formation of fibrils in the droplets. We observe changes for two major absorption features: an increase of the cavity-wrap band around 150 cm^−1^ and a decrease of the peak centered at 500 cm^−1^ with a narrowing of its width, representing a decrease in bound hydration water forming hydrogen bonds with the protein. (*b*) The schematic (not generated from computational simulation) illustrates the transformation of droplets into fibrils, a process referred to as liquid-to-solid phase separation (LSPS). During fibril formation, the proteins form densely packed parallel β sheet structures. This is accompanied by “drying,” i.e., a loss of bound water and the exposure of the less hydrophilic regions of the fibrils to the surrounding water. Consequently, the minimum around 150 cm^−1^ is no longer visible, and we also observe a decrease in the band centered at 500 cm^−1^ along with a line narrowing.

With increasing time, the droplets evolve into fibrils and solidify on the ATR crystal. Fibril formation is associated with the appearance of parallel β sheet structures, as deduced from the results of FT-IR spectroscopic and AFM measurements (Fig. 1 *c*). This indicates that protein-protein interactions are outweighing protein-water interactions. As a consequence, we expect a loss of bound water and a “dewetting” of the protein interface. The changes in the THz spectra in this later phase of fibril formation are plotted in Fig. 4 *a*. Indeed, we observed a narrowing of the linewidth centered at 500 cm^−1^, which serves as an indicator of the decrease in the coupling between the protein and the hydration water during this morphological transformation. Fig. 4 *b* depicts a schematic summary of the underlying molecular processes that is in line with this hypothesis. While the proteins adopt a tightly packed parallel β sheet conformation, the exposure of hydrophilic side chains to water is minimized. Thus, upon liquid-to-solid phase separation (LSPS), the bound water population (represented in blue) is no longer retained. Interestingly, this does affect less the “wrap water” (in red color) since most of the wrapped water population was already released from the droplets upon LLPS.

**Figure 5:**
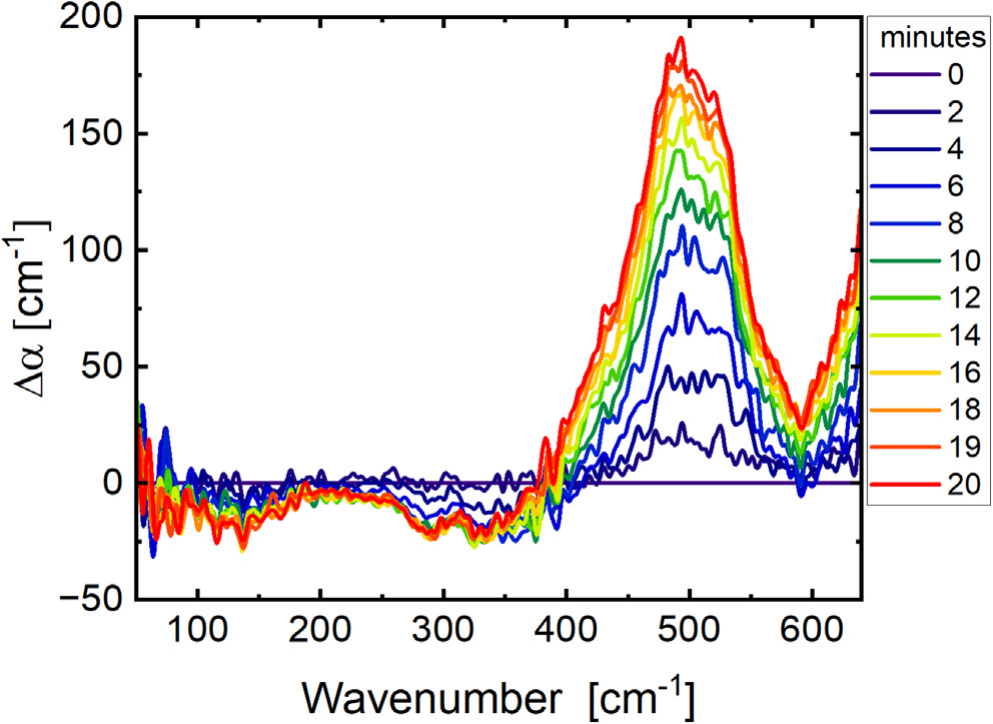
Time series of ATR absorption difference spectra of the polylysine protein (1 mg/mL) solvated in an ATP solution (0.71 mg/mL). We plot Δ*α*, i.e., the change in THz absorption after a time delay (*t*) and the initial absorption spectra (*t* = 0).

For comparison, we also recorded THz spectra using a mixture of polylysine and ATP (see Fig. 5). It is well established that a polylysine-ATP solution gives rise to the formation of liquid-liquid phase-separated droplets; however, it does not form any solid aggregates (30), and the droplets stay intact for a long time. Within the first 20 min, we observed a decrease in Δ*α* in the frequency range between 100 and 150 cm^−1^, e.g., a loss of the cavity-wrap water population hydrating hydrophobic parts of the polylysine as well a continuous increase at 500 cm^−1^, e.g., beyond 20 min, in this case, the spectrum remains unchanged. This observation supports our previous assumption that LLPS goes along with a loss of cavity wrap.

For the polylysine-ATP mixture, the spectrum remained unchanged after 20 min, unlike the investigated ACC_1–13_K_24_-ATP sample, which showed a decrease in the coupling between the protein and the hydration water at 500 cm^−1^. Based on the differences in the spectra, we propose that fibril formation involves a loss of bound water. This is a consequence of the favorable enthalpic protein-protein intermolecular interactions compared to the protein-water interactions. Whereas for LLPS, a subtle balance between a loss of entropically unfavorable water hydrating hydrophobic groups and an enthalpically favorable retaining of water hydrating the hydrophilic groups is mandatory to compensate the unfavorable entropic protein-protein condensation. In the case of LSPS, strong protein-protein interactions are the main thermodynamic driver, i.e., are the dominant term for changes in the Gibbs free energy. We want to point that protein-protein interactions are less temperature sensitive than protein water interactions. Thus, LSPS is irreversible and cannot be easily tuned with temperature.

The total solvation free energy of a protein (or any other solute) is given by the sum of the free energy terms associated with these two steps, i.e.,

(3) $$\Delta G_{\text{solv}}=\Delta G_{\text{cavity}}+\Delta G_{\text{bound}}$$

Following previous THz-calorimetry studies, we can express the free energy difference between the final and initial states of LLPS and LSPS (23):

(4) $$\Delta G_{\text{LLPS}/\text{LSPS}}=\Delta \alpha_{\text{wrap}}\left(\Delta H_{\text{wrap}}-T\Delta S_{\text{wrap}}\right)+\Delta \alpha_{\text{bound}}\left(\Delta H_{\text{bound}}-T\Delta S_{\text{bound}}\right)+\Delta G_{p-p}$$

where Δ*G*_p-p_ denotes the partial contributions due to protein-protein interactions, which are mostly enthalpic.

**Figure 6:**
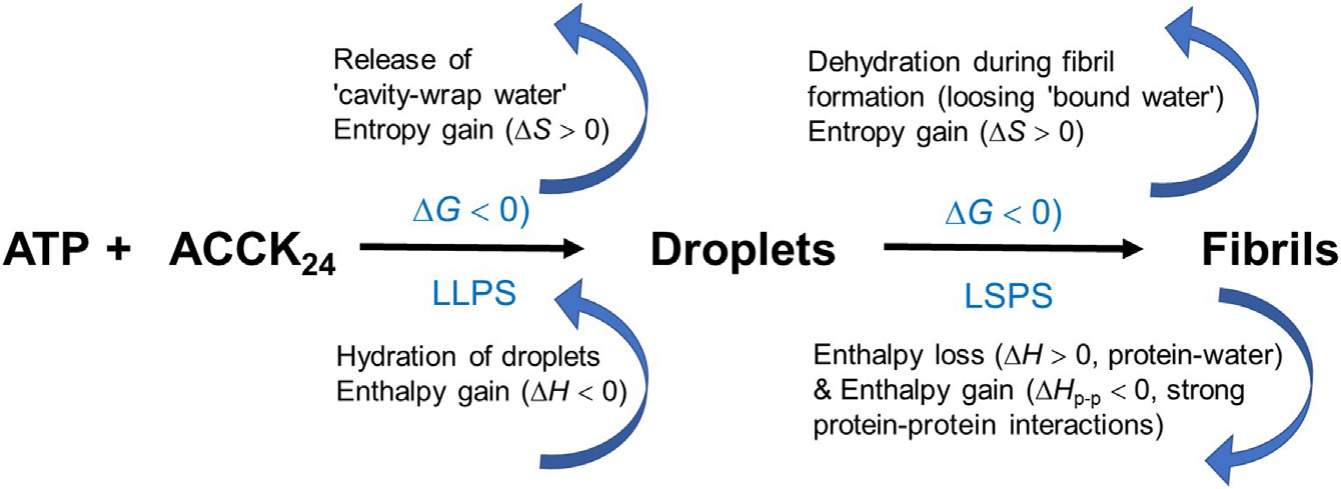
Solvation thermodynamics: the schematic elucidates the mechanisms underlying droplet formation. We propose that the following two thermodynamic driving forces contribute: 1) an increase in entropy from the release of entropically unfavorable cavity-wrap hydration water and 2) an enthalpic gain by the retained bound water upon protein condensation. The evolution from droplet to fibril formation is further facilitated by an entropy increase, resulting from the expulsion of water molecules initially confined in the droplet, and an overcompensation of the enthalpic unfavorable loss of bound water molecules compared to the enthalpic gain by protein-protein interactions, mostly due to interstrand H-bonding.

During droplet formation as shown in Fig. 3, we observed a release of cavity-wrap water, thus yielding Δ*S*_wrap_ ˃ 0. This is the dominant driving force for LLPS. Simultaneously, retaining of the bound water helps to minimize enthalpy changes. Upon subsequent fibril formation (LSPS), the THz spectra revealed a loss of bound hydration water as part of the dewetting. The coupling to the water network is weaker, as indicated by a smaller linewidth. This results in a positive change in entropy ($\Delta S_{\text{bound}}$ > 0). Therefore, solvation entropy favors both LLPS as well as the subsequent fibrillization process, although via contributions from two distinct hydration mechanisms: hydrophobic cavity-wrap and hydrophilic interactions, respectively. The solvation entropy driving force plays an essential role in offsetting the unfavorable conformational entropy contribution expected from protein-protein interactions (that we cannot quantify) due to initial crowding and subsequent aggregation and fibrillization. The process of fibril formation is accompanied by a positive/unfavorable change in solvation enthalpy (Δ*H*_bound_ > 0) due to a loss of protein-water interactions, which is outweighed by favorable protein-protein interactions (Δ*H*_p-p_ < 0). Attractive protein-protein interactions, particularly between ATP and ACC_1–13_K_24_, are present in the transition to a densely packed β sheet structure, as incorporated in Δ*G*_p-p_ in Eq. 4. Our results are presented in a visualization shown in Fig. 6.

### Conclusion

In this study, we present the combined results of our study investigating the solvation driving forces during liquid droplets and subsequent fibril formation in mixtures of ATP and ACC_1–13_K_24_ peptide. We followed the LLPS and LSPS processes using microscopy, FT-IR, and THz spectroscopic measurements. We conclude that droplet formation is accompanied by a release of entropically favorable cavity-wrap water and an enthalpy contribution due to the retention of hydrogen bond water in the droplet. Whereas favorable enthalpic protein-protein interactions are a “necessary precondition,” the changes in the hydration enthalpy and entropy allow a fine-tuning of the process and are decisive for protein condensation and fibril formation. Fibril formation is accompanied by an expulsion of water molecules initially confined in the droplet and an overcompensation of the enthalpic unfavorable loss of bound water molecules by the enthalpic gain due to intermolecular protein-protein interactions in the pleated β sheet structure.

## Acknowledgments

The authors acknowledge funding from the 10.13039/501100001659Deutsche Forschungsgemeinschaft (DFG, German Research Foundation) under Germany's Excellence Strategy – EXC 2033 – project number 390677874-RESOLV. We acknowledge financial support by 10.13039/501100000781European Research Council (ERC) Advanced Grant 695437 THz-Calorimetry. This work is carried out in the “ZEMOS - Home of Solvation Science” funded by the German Federal Ministry of Education and Research 10.13039/501100002347BMBF and by the Ministry of Culture and Research of Nord Rhine-Westphalia (NRW) MKW.

## Author contributions

S.B. and R.D. performed the experiments. R.D. carried out the synthesis of the peptide. S.B., R.D., S.P., G.S., R.W., and M.H. analyzed the data and wrote the manuscript. R.R.S. assisted in some of the measurements. The overall project was designed and conducted under the supervision of M.H. and R.W.

## Declaration of interests

The authors declare no competing interests.
